# The Relationship between Adiponectin and Left Ventricular Mass Index Varies with the Risk of Left Ventricular Hypertrophy

**DOI:** 10.1371/journal.pone.0070246

**Published:** 2013-07-24

**Authors:** Yonggu Lee, Bae Keun Kim, Young-Hyo Lim, Mi Kyung Kim, Bo Youl Choi, Jinho Shin

**Affiliations:** 1 Department of Cardiology, Hanyang University Hospital, Seoul, Republic of Korea; 2 Preventive Medicine, Hanyang University College of Medicine, Seoul, Republic of Korea; 3 Cardiology Clinic, Myungji St. Mary’s Hospital, Seoul, Republic of Korea; Scuola Superiore Sant'Anna, Italy

## Abstract

**Background:**

Adiponectin directly protects against cardiac remodeling. Despite this beneficial effect, most epidemiological studies have reported a negative relationship between adiponectin level and left ventricular mass index (LVMI). However, a positive relationship has also been reported in subjects at high risk of left ventricular hypertrophy (LVH). Based on these conflicting results, we hypothesized that the relationship between serum adiponectin level and LVMI varies with the risk of LVH.

**Methods:**

A community-based, cross-sectional study was performed on 1414 subjects. LVMI was measured by echocardiography. Log-transformed adiponectin levels (Log-ADPN) were used for the analysis.

**Results:**

Serum adiponectin level had a biphasic distribution (an increase after a decrease) with increasing LVMI. Although Log-ADPN did not correlate with LVMI, Log-ADPN was modestly associated with LVMI in the multivariate analysis (*β* = 0.079, *p* = 0.001). The relationship between adiponectin level and LVMI was bidirectional according to the risk of LVH. In normotensive subjects younger than 50 years, Log-ADPN negatively correlated with LVMI (*r* = −0.204, *p* = 0.005); however, Log-ADPN positively correlated with LVMI in ≥50-year-old obese subjects with high arterial stiffness (*r* = 0.189, *p* = 0.030). The correlation coefficient between Log-ADPN and LVMI gradually changed from negative to positive with increasing risk factors for LVH. The risk of LVH significantly interacted with the relationship between Log-ADPN and LVMI. In the multivariate analysis, Log-ADPN was associated with LVMI in the subjects at risk of LVH; however, Log-ADPN was either not associated or negatively associated with LVMI in subjects at low risk of LVH.

**Conclusion:**

Adiponectin level and LVMI are negatively associated in subjects at low risk of LVH and are positively associated in subjects at high risk of LVH. Therefore, the relationship between adiponectin and LVMI varies with the risk of LVH.

## Introduction

Adiponectin is a 30-kDa peptide hormone almost exclusively secreted by adipose tissue [1]. Adiponectin mediates insulin sensitivity in peripheral tissues and has protective effects against atherosclerosis [1], [2]. Adiponectin may also have cardio-protective effects by reducing fibrosis and apoptosis, improving energy metabolism and preventing myocyte hypertrophy [3]. Shibata et al. [4] reported that adiponectin reversed cardiac hypertrophy in wild-type and transgenic obese mice by directly influencing the fat and glucose metabolism in cardiac myocytes via the adenosine monophosphate activated-protein kinase signaling pathway. Several epidemiologic and clinical studies have also shown a link between low adiponectin levels and left ventricular hypertrophy (LVH) [5]–[8].

However, unlike animal studies, epidemiologic and clinical studies have often given conflicting results. In a study with kidney transplant patients, low adiponectin was not associated with the occurrence of LVH [9]. However, a recent cohort study found that adiponectin level was positively associated with left ventricular mass index (LVMI) after multivariate adjustments in subjects with hypertension and insulin resistance [10]. Studies in patients on dialysis have also shown a positive relationship between adiponectin level and LVMI [11], [12]. Moreover, adiponectin is frequently increased in congestive heart failure (CHF) and is considered a strong predictor of mortality in patients with CHF [13]–[15]. Whether adiponectin plays a protective or detrimental role in CHF remains unclear. However, considering its beneficial effects on the cardiovascular system, the increase in adiponectin in CHF might be part of a compensatory mechanism [15]. Hypertension, insulin resistance and dialysis are well-known risk factors for LVH, which is a key feature of cardiac remodeling in CHF. These findings suggest that the relationship between adiponectin and LVMI varies with the risk of LVH. Therefore, we investigated the difference in the relationship between adiponectin and LVMI in subjects with different severities and risks of LVH.

## Subjects and Methods

### Subjects

The Institutional Review Board of Hanyang University approved the design and procedures of this study. The Yangpyeong Cohort Study is a longitudinal, community-based cohort study evaluating the determinants of cardiovascular disease [16]. Yangpyeong County is a rural area located 45 km east of Seoul, the capital of South Korea. In this study, 5 of the 12 districts were selected as study areas, and residents over 40 years old were informed of the study. Between 2007 and 2009, 1,841 voluntary participants were recruited. Written informed consent was obtained from all of the participants prior to starting the study. Of the 1,841 participants, 1,414 were enrolled after completing a questionnaire about social and demographic information. Individuals with diabetes who were on insulin or oral hypoglycemic agents and individuals with severe cardiovascular diseases, decreased left ventricular systolic function or major organ dysfunction were excluded. The individuals also underwent a clinical examination and blood tests. The clinical examination included measurements of height, weight, blood pressure, brachial–ankle pulse wave velocity (baPWV) and transthoracic echocardiography. The blood tests included glucose, insulin, and lipid profiles and adiponectin level. The body mass index (BMI) was calculated using the following equation: BMI = weight/height^2^ (kg/m^2^). Obesity was defined as a BMI ≥25 kg/m^2^ according to the guidelines for Asian populations [17]. Healthy subjects were defined as subjects without any of following conditions: hypertension, impaired fasting glucose, obesity and LVH. All the questionnaires and examinations were conducted by trained personnel using a structured questionnaire and protocol.

### Blood Tests

Blood samples were collected in the morning after at least 8 hours of fasting. Serum glucose, creatinine, total cholesterol, triglycerides and high-density lipoprotein (HDL) cholesterol were measured using an ADVIA1650 Automatic Analyzer (Siemens, New York, USA). Non-HDL cholesterol was calculated from the total cholesterol by subtracting HDL. The estimated glomerular filtration rate (eGFR) was calculated using the Modification of Diet in Renal Disease study equation. An immunoradiometric assay with an insulin RIA kit (Biosource, Belgium) was used to measure serum insulin. The concentration of total adiponectin was obtained by a radioimmunoassay using a human adiponectin RIA kit (LINCO Research, Inc., Billerica, MA, USA). The homeostatic model assessment of insulin resistance (HOMA-IR) was calculated using the following equation:
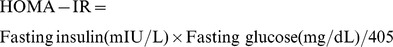



Log-transformed adiponectin (Log-ADPN) and Log-transformed HOMA-IR (Log-HOMA-IR) were used in our analysis to stabilize the distribution. High and low insulin resistance was defined as the first and fourth quartile of Log-HOMA-IR, respectively. Impaired fasting glucose was defined as fasting glucose ≥110 mg/dL.

### Echocardiography and Measurement of Brachial-ankle Pulse Wave Velocity

Transthoracic 2D and guided M-mode echocardiography were performed on all of the subjects by a single cardiologist using a single echocardiography machine (ACUSON S2000, Siemens, USA) with a 2.5/2.0 MHz transducer. All of the measurements were collected using the standard methods specified in the guidelines of the American Society of Echocardiography [18]. M-mode images were obtained from the parasternal long-axis view at the level of or just below the anterior mitral leaflet. All of the M-mode measurements for calculating left ventricular mass (LVM) were made during end-diastole. The interventricular septal thickness (IVSd), left ventricular internal diameter (LVIDd) and left ventricular posterior wall thickness (LVPWd) were measured using the leading edge-to-leading edge method. LVM and LVMI were calculated using the following equations:
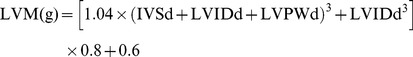


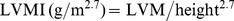



Mild LVH was defined as an LVMI ≥49 g/m^2.7^ in males and an LVMI ≥45 g/m^2.7^ in females, and moderate to severe LVH was defined as an LVMI ≥56 g/m^2.7^ in males and an LVMI ≥52 g/m^2.7^ in females, according to the recommendations of the American Society of Echocardiography [18]. Decreased systolic function was defined as an ejection fraction <55% measured in M-mode.

Measurements of baPWV were collected from both sides using conventional methods with an automatic waveform analyzer (VP-2000, Colin Medical Technology Co., Komaki, Japan) after the subjects rested for at least 5 minutes in the supine position. Systolic blood pressure (SBP), diastolic blood pressure (DBP), pulse rate and electrocardiogram were recorded simultaneously with baPWV. The average baPWV from both sides was used for the analysis. Low arterial stiffness was defined as the first quartile of baPWV, and high arterial stiffness was defined as the fourth quartile of baPWV.

### Statistical Analysis

The subjects were stratified into four groups according to the severity of LVH: healthy subjects, no LVH, mild LVH and moderate to severe LVH. A one-way ANOVA with Bonferroni correction (Kruskal-Wallis test in case the data failed tests for homogeneity of variance) was performed to compare continuous variables (e.g., age, SBP, DBP, BMI, baPWV, LVMI, Log-ADPN and Log-HOMA-IR). The chi-square test was used to compare the categorical variables (e.g., gender, impaired fasting glucose and hypertension). Pearson’s correlations were performed to identify the simple correlations between LVMI and the other variables. Stepwise multiple linear regression analyses were performed with the variables that correlated with LVMI to identify the variables that independently influenced LVMI. Hypertension, pulse pressure, impaired fasting glucose and baPWV were not included in the regression analysis because of their collinearity with other variables, such as age, SBP and BMI. Pearson’s correlations were performed to identify the relationship between Log-ADPN and LVMI in various groups with different risks of LVH. Risk factors for LVH were determined based on the results of the correlation analyses. A logistic regression analysis was used to determine the odds ratios (ORs) of risk factors for LVH and to produce the risk scores for LVH. The interactions of each risk factor and the risk scores for LVH with the relationship between Log-ADPN and LVMI were tested using multiple linear regression analyses with the enter method. Log-ADPN and the risk scores for LVH were centered around their means to avoid collinearity. A stepwise multiple linear regression analysis was performed using the variables related to LVMI to identify the independent variables affecting LVMI in each group. The results are expressed as the mean ± SD. Statistical significance was defined as *p*<0.05. All the statistical analyses were performed using SPSS 20.0 for Windows (IBM, New York, USA).

## Results

### Baseline Characteristics of the Study Population

The mean age of the study population was 61.14±10.35 years, and 40.2% of all subjects were male. Impaired fasting glucose was present in 22.6% of the subjects, and hypertension was present in 44.4% of the subjects. The mean SBP and DBP were 124.78±17.67 mmHg and 80.40±10.54 mmHg, respectively. The mean BMI was 24.97±3.23 kg/m^2^, and the mean eGFR was 69.02±11.63 mL/min/1.73 m^2^. The mean HOMA-IR was 2.63±1.61. The mean baPWV was 1,605.76±336.61 cm/s. Among the 1,414 subjects, 274 healthy subjects were found. The mean adiponectin level in all of the subjects combined was lower than that in the healthy subjects (8,169.0±4,905.2 ng/mL vs. 9081.2±4713.5 ng/mL, *p*<0.001). LVM and LVMI in all the subjects combined were higher than those in the healthy subjects (155.7±39.7 g vs. 129.4±28.5 g, *p*<0.001 and 45.7±11.5 g/m^2.7^ vs. 36.3±6.1 g/m^2.7^, *p*<0.001, respectively). Mild LVH was detected in 268 subjects, and moderate to severe LVH was detected in 306 subjects.

The baseline characteristics of the four groups according to the severity of LVH (healthy subject group, no LVH, mild LVH and moderate to severe LVH) are shown in Table 1. The mean age, SBP, DBP, waist-hip ratio, BMI, eGFR, total and non-HDL cholesterol, baPWV, LVM and HOMA-IR in healthy subjects were lower than those in the no LVH group, whereas adiponectin and Log-ADPN in healthy subjects were higher than in the no LVH group. The frequencies of female gender, impaired fasting glucose and hypertension increased with the severity of LVH. The mean age, SBP, BMI, baPWV, LVM and LVMI also increased with the severity of LVH, whereas DBP, waist-hip ratio, total and non-HDL cholesterol and HOMA-IR did not change among the no LVH, mild LVH and moderate to severe LVH groups. Adiponectin and Log-ADPN in the moderate to severe LVH group were higher than those in the no LVH group.

**Table 1:** Clinical characteristics of the study population.

	Healthy subjects†	No LVH	Mild LVH	Moderate to severe LVH	
	(n = 274)	(n = 566)	(n = 268)	(n = 306)	*p*
Age (Years)	56.7±10.7	61.0±10.3*	61.8±10.0*	64.7±8.9***	<0.001
Male, n (%)	118 (43.1)	293 (51.8)	87 (32.5)	71 (23.2)	<0.001
IFG, n (%)	–	167 (28.6)	67 (25.0)	91 (29.7)	<0.001
Hypertension, n (%)	–	296 (52.3)	134 (50.0)	198 (64.7)	<0.001
SBP (mmHg)	112.6±11.4	126.0±16.2*	127.5±19.5*	131.1±18.8**	<0.001
DBP (mmHg)	75.3±7.1	81.7±10.5*	81.6±11.4*	81.7±10.5*	<0.001
Waist-hip ratio	0.89±0.06	0.93±0.06*	0.92±0.06*	0.93±0.06*	<0.001
BMI (kg/m^2^)	22.3±3.1	24.0±3.1*	25.3±2.8*	26.5±3.4**	<0.001
eGFR (mL/min/1.73 m^2^)	73.7±8.8	67.7±11.9*	69.3±12.1*	67.1±11.9*	<0.001
Total cholesterol (mg/dL)	189.7±33.0	201.8±38.8*	200.5±37.7*	201.5±36.3*	<0.001
Non-HDL cholesterol (mg/dL)	152.0±35.8	152.0±35.8*	155.0±34.5*	155.1±35.2*	<0.001
Average baPWV (cm/s)	1443.8±257.5	1649.3±343.7*	1600.0±308.4*	1675.3±336.6***	<0.001
LVM (g)	129.4±28.5	137.0±30.0*	165.7±29.2**	198.0±35.1***	<0.001
LVMI (g/m^2.7^)	36.3±6.1	39.3±5.5*	49.6±2.7**	62.4±8.2***	<0.001
HOMA-IR	1.9±0.8	2.8±1.8*	2.7±1.6*	2.9±1.7*	<0.001
Adiponectin (ng/mL)	9081.2±4713.5	7349.2±4786.4*	8162.6±5139.6	8874.0±4805.4**	<0.001
Log-ADPN	8.97±0.56	8.71±0.63*	8.83±0.59**	8.94±0.57***	<0.001

The data are shown as the mean ± SD or n (%).

* different from the value in the healthy subject group.

** different from the value in the no LVH group.

*** different from the value in the mild LVH group.

† Subjects without hypertension, obesity (BMI ≥25 kg/m^2^), IFG and LVH.

IFG, impaired fasting glucose; SBP, systolic blood pressure; DBP, diastolic blood pressure; BMI, body mass index; eGFR, estimated glomerular filtration rate; HDL, high-density lipoprotein; baPWV, brachial-ankle pulse wave velocity; LVM, left ventricular mass; LVMI, left ventricular mass index; HOMA-IR, homeostatic model assessment of insulin resistance; Log-HOMA-IR, log-transformed HOMA-IR; Log-ADPN, log-transformed adiponectin.

### Relationship between the Log-ADPN and LVMI

**Figure 1:**
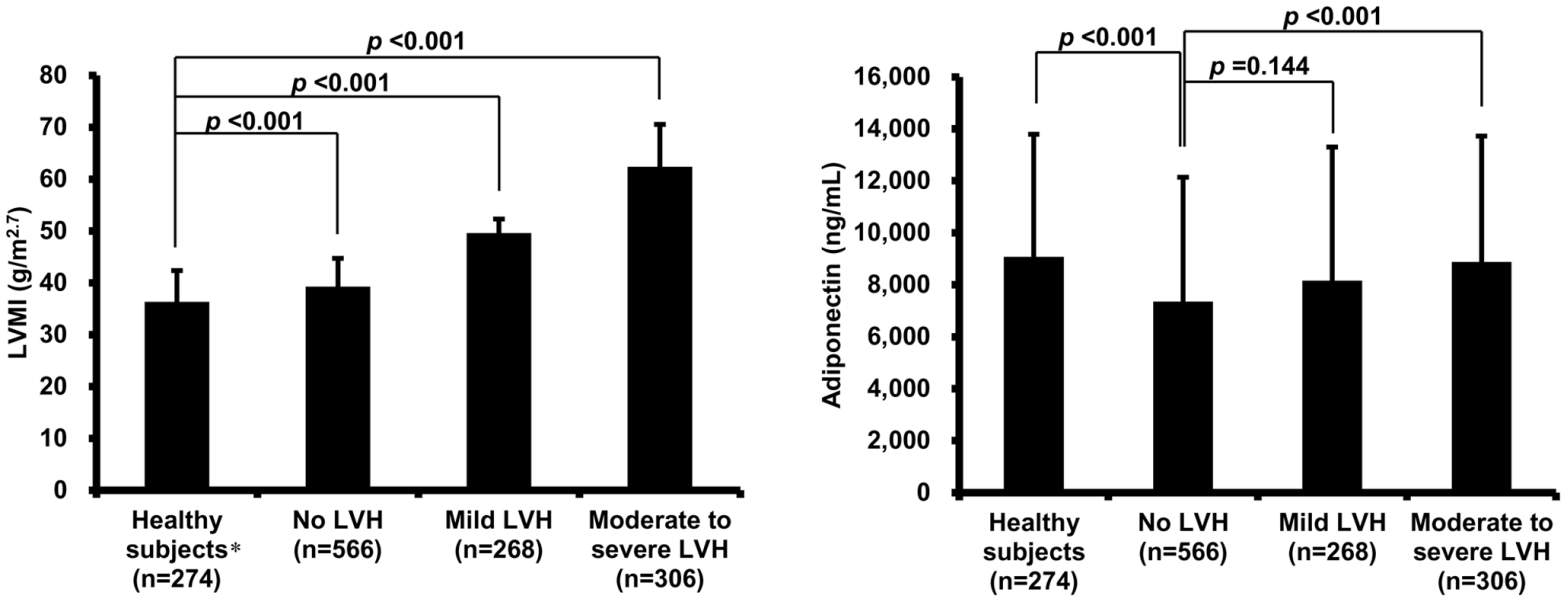
The adiponectin level and LVMI according to the severity of LVH. The bars and whiskers indicate the means and standard deviations. * Subjects without hypertension, obesity (body mass index ≥25 kg/m^2^), impaired fasting glucose and LVH. LVMI increases with the severity of LVH. Adiponectin in the healthy subjects is higher than in subjects without LVH, whereas adiponectin in subjects without LVH is lower than in subjects with moderate to severe LVH. LVMI, left ventricular mass index; LVH, left ventricular hypertrophy.

LVMI gradually increased with the severity of LVH. However, the adiponectin level showed a biphasic distribution; a decrease in the no LVH group compared to the healthy subjects and an increase in the moderate to severe LVH group compared to the no LVH group (Figure 1). LVMI positively correlated with BMI, SBP, age and Log-HOMA-IR, negatively correlated with male gender and did not correlate with Log-ADPN (Table 2). In a stepwise multiple linear regression analysis, LVMI was positively associated with BMI, age, SBP and Log-ADPN and was not associated with Log-HOMA-IR or male gender. BMI was the strongest determinant of LVMI, whereas Log-ADPN was only a modest determinant of LVMI (*β* = 0.079, *p* = 0.001) (Table 2). LVMI and Log-ADPN were negatively correlated in the no LVH group and mild LVH group (*r* = −0.125, *p* = 0.003 and *r* = −0.273, *p*<0.001, respectively), but they did not correlate in the healthy subject group or moderate to severe LVH group (*r* = −0.038, *p*>0.05 and *r* = 0.045, *p*>0.05, respectively).

**Table 2:** The relationship between LVMI and other variables.

	Simple correlation		Multiple linear regression*	
	*r*	*p*	*β*	*p*
BMI	0.367	<0.001	0.388	<0.001
SBP	0.243	<0.001	0.160	<0.001
Age	0.228	<0.001	0.227	<0.001
Log-HOMA-IR	0.146	<0.001	–	–
Male	−0.091	0.001	–	–
Log-ADPN	0.033	*NS*	0.079	0.001

* The regression analysis was performed using the stepwise method.

LVMI, left ventricular mass index; BMI, body mass index; SBP, systolic blood pressure; Log-HOMA-IR, log-transformed homeostatic assessment of insulin resistance; Log-ADPN, log-transformed adiponectin; *NS*, not significant.

### Correlation between Log-ADPN and LVMI in Groups with or without Risk Factors for LVH

In the univariate logistic regression analysis, the risk factors for LVH were hypertension (OR 2.52, *p*<0.001), obesity (OR 2.96, *p*<0.001), age ≥50 (OR 2.25, *p*<0.001) and high arterial stiffness (OR 1.89, *p*<0.001). Log-ADPN and LVMI negatively correlated in subjects <50 years old, and this negative correlation was stronger in normotensive subjects <50 years old. Log-ADPN and LVMI positively correlated in subjects with hypertension, obesity or high arterial stiffness, whereas they did not correlate in subjects without hypertension or obesity or in subjects with low arterial stiffness. The positive correlation between Log-ADPN and LVMI was stronger when multiple risk factors for LVH were present (Table 3).

**Table 3:** Correlations between Log-ADPN and LVMI.

	Group criteria	n	*r*	*p*
Groups at low risk of LVH	Normotension+Age <50 years	187	−0.204	0.005
	Age <50 years	249	−0.150	0.018
	No obesity *	781	−0.046	*NS*
	Normotensive	798	−0.026	*NS*
	Low arterial stiffness†	353	−0.015	*NS*
Groups with a single risk factor for LVH	Hypertension	628	0.118	0.003
	Obesity	633	0.140	<0.001
	High arterial stiffness^‡^	353	0.149	0.005
Groups with multiple risk factors for LVH	Obesity+Hypertension	341	0.159	0.003
	Obesity+High arterial stiffness	138	0.179	0.036
	Obesity+High arterial stiffness+Age ≥50 years	132	0.189	0.030

* Body mass index <25 kg/m^2^.

† and ^‡^ The lowest and highest quartiles, respectively, for the brachial-ankle pulse wave velocity.

Log-ADPN, log-transformed adiponectin; LVMI, left ventricular mass index; LVH, left ventricular hypertrophy; *NS*, not significant.

### Interaction of Risk Factors for LVH with the Relationship between Log-ADPN and LVMI

**Figure 2:**
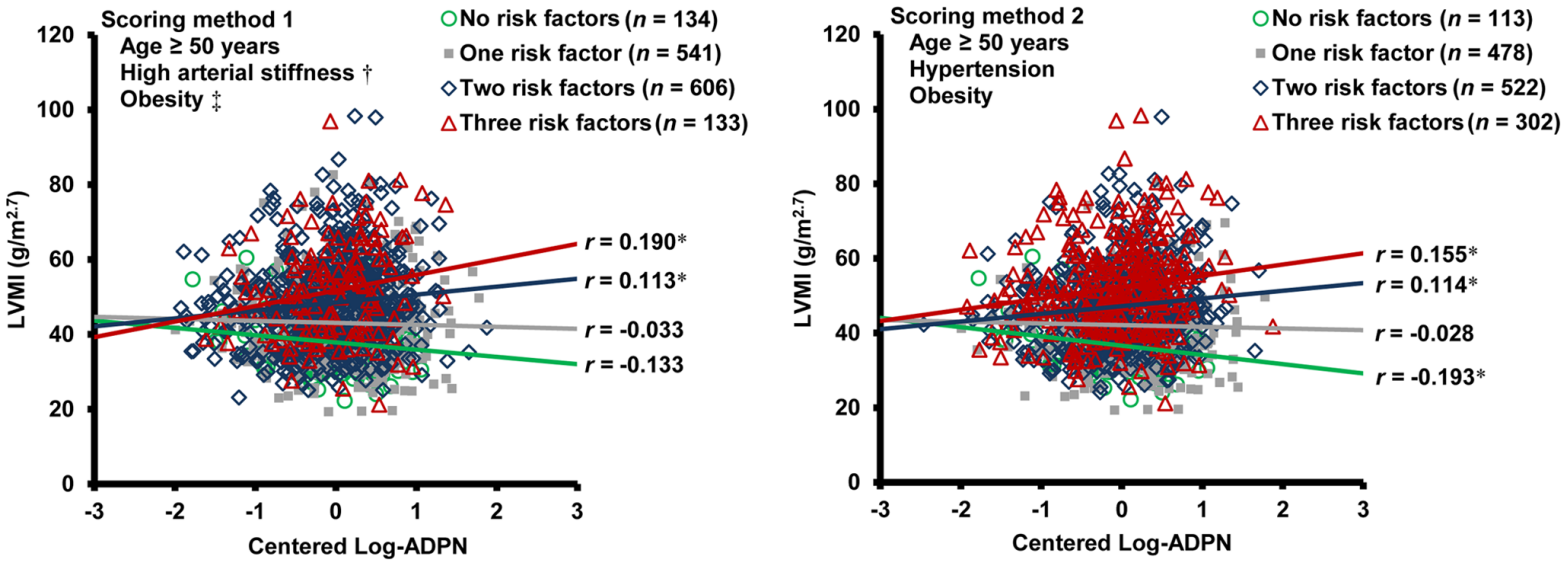
Variations in the correlation between LVMI and Log-ADPN according to the risk of LVH. * *p*<0.05. † The highest quartile for the brachial-ankle pulse wave velocity. ‡ BMI ≥25 kg/m^2^. Log-ADPN is centered around the mean. The direction of the correlations between adiponectin level and LVMI gradually changes from negative to positive with the number of LVH risk factors in both scoring methods. LVMI, left ventricular mass index; Log-ADPN, log-transformed adiponectin; LVH, left ventricular hypertrophy.

Two different scoring methods for assessing the risk of LVH were used. In scoring method 1, the risk factors were age ≥50 years, high arterial stiffness and obesity. In scoring method 2, the risk factors were age ≥50 years, hypertension and obesity. LVMI gradually increased with the number of risk factors. The mean adiponectin level in subjects with one risk factor was higher than that in the other subjects, but there were no differences among mean adiponectin levels in the other subject groups. The OR gradually increased with the number of risk factors, and the increments were roughly similar between consecutive groups based on the number of risk factors (Table 4). The correlations between the centered Log-ADPN and LVMI varied with the number of LVH risk factors in both scoring methods (Figure 2). In subjects without risk factors, Log-ADPN negatively correlated with LVMI (*r* = −0.193, *p* = 0.041 in scoring method 2). However, the correlation between Log-ADPN and LVMI gradually changed from negative to positive with an increasing number of risk factors. The positive correlation between Log-ADPN and LVMI was strongest in subjects with three risk factors for LVH based on scoring methods 1 and 2 (*r* = 0.190, *p* = 0.028 and *r* = 0.155, *p* = 0.007, respectively). Age ≥50, hypertension, obesity and high arterial stiffness significantly interacted on the relationship between Log-ADPN and LVMI. There were also significant interactions of the number of LVH risk factors on the relationship between Log-ADPN and LVMI in both scoring methods (Table 5).

**Table 4:** Risk stratification for LVH.

	Risk factor	No. of risk factors	LVMI (g/m^2.7^)	Adiponectin (ng/mL)	OR for LVH*	CI	*p*
Scoring method 1	Age ≥50 years	0	36.7±7.4	7651.7±3822.9	–	–	–
	High arterial stiffness †	1	42.1±9.4	8871.3±5256.8	2.80	1.67–4.70	<0.001
	Obesity ‡	2	47.1±11.0	7878.7±4731.9	6.09	3.66–10.16	<0.001
		3	52.1±12.4	7751.6±4882.0	9.14	5.03–16.59	<0.001
Scoring method 2	Age ≥50 years	0	37.9±8.2	7705.6±3777.7	–	–	–
	Hypertension	1	43.1±9.8	8734.0±5270.6	3.46	1.80–6.66	<0.001
	Obesity	2	48.4±11.7	7848.2±4817.0	7.92	4.15–15.10	<0.001
		3	51.6±13.1	7798.8±4588.6	16.42	8.45–31.92	<0.001

Data for LVMI and adiponectin are shown as the mean ± SD.

The odds ratios gradually increase with the number of risk factors.

* In comparison to the subjects with no risk factors.

† Body mass index ≥25 kg/m^2^_._

‡ The highest quartile of brachial-ankle pulse wave velocity.

LVH, left ventricular hypertrophy; LVMI, left ventricular mass index; OR, odds ratio; CI, confidence interval.

**Table 5:** Interactions of the risk factors for LVH with the relationship between Log-ADPN and LVMI.

	*β*	*p*
Age ≥50 years		
Centered variables	0.187	<0.001
Centered Log-ADPN	0.012	0.645
Interaction*	0.064	0.016
Hypertension		
Centered variables	0.254	<0.001
Centered Log-ADPN	0.039	0.130
Interaction	0.071	0.006
Obesity†		
Centered variables	0.332	<0.001
Centered Log-ADPN	0.087	0.001
Interaction	0.049	0.050
High arterial stiffness‡		
Centered variables	0.092	<0.001
Centered Log-ADPN	0.033	0.216
Interaction	0.073	0.006
Scoring method 1		
Centered variables	0.339	<0.001
Centered Log-ADPN	0.047	0.060
Interaction	0.097	<0.001
Scoring method 2		
Centered variables	0.400	<0.001
Centered Log-ADPN	0.057	0.021
Interaction	0.087	<0.001

* (Centered Log-ADPN) × (Centered variables).

† Obesity was defined as BMI ≥25 kg/m^2^.

‡ The highest quartile for the brachial-ankle pulse wave velocity.

Log-ADPN, age ≥50, hypertension, arterial stiffness, scoring method 1 and scoring method 2 were centered around their means.

LVMI, left ventricular mass index; LVH, left ventricular hypertrophy; Log-ADPN, log-transformed adiponectin.

### Stepwise Multiple Regression Analysis for LVMI in Subjects with a High or Low Risk of LVH

Log-ADPN was not associated with LVMI in subjects without hypertension or obesity or in subjects with low arterial stiffness. However, Log-ADPN was positively associated with LVMI in subjects with hypertension (*β* = 0.095, *p* = 0.020), obesity (*β* = 0.121, *p* = 0.001) or high arterial stiffness (*β* = 0.192, *p*<0.001) (Table 6).

**Table 6:** Stepwise multiple linear regression analysis for the determinants of LVMI in subjects at high or low risk of LVH.

	Low risk of LVH			High risk of LVH		
	Normotension	No obesity	Low arterial stiffness	Hypertension	Obesity	High arterial stiffness
Age	0.280†	0.259†	0.301†	0.156†	0.232†	–
BMI	0.394†	0.206†	0.335†	0.337†	0.230†	0.306†
SBP	0.084*	0.210†	0.203†	0.117*	0.127*	0.149*
Male	–	−0.074*	–	−0.105*	–	–
Log-HOMA-IR	–	–	–	–	–	–
Log-ADPN	–	–	–	0.095*	0.121*	0.192†

* *p*<0.05,

† *p*<0.001.

– The numbers are the standardized β weights of the determinants.

– Obesity is defined as a body mass index ≥25 kg/m^2^.

– Low and high arterial stiffness are defined as the lowest and the highest quartiles of the brachial-ankle pulse wave velocity, respectively.

LVMI, left ventricular mass index; SBP, systolic blood pressure; Log-HOMA-IR, log-transformed homeostatic assessment of insulin resistance; Log-ADPN, log-transformed adiponectin.

## Discussion

### Main Findings

We found that the relationship between Log-ADPN and LVMI varied with the risk of LVH. The crude correlation between Log-ADPN and LVMI was not significant in the entire study population, whereas a modest positive association between adiponectin level and LVMI was observed in the stepwise multiple linear regression analysis. This could be explained by the biphasic distribution of serum adiponectin level and Log-ADPN to increasing LVMI. In subjects at low risk of LVH, Log-ADPN was either negatively or not significantly associated with LVMI. In contrast, for subjects at high risk of LVH, Log-ADPN positively correlated with LVMI. The positive relationship between Log-ADPN and LVMI was stronger with an increasing number of LVH risk factors. This trend was also supported by the interaction of the number of LVH risk factors with the relationship between Log-ADPN and LVMI and the positive association between Log-ADPN and LVMI in multiple linear regression analyses, which was only observed in subjects at high risk of LVH. These two different relationships between adiponectin level and LVMI indicate different mechanisms that influence adiponectin production in subjects at different risk of LVH.

### Relationship between Plasma Adiponectin Concentration and LVMI

Low serum adiponectin is common in subjects with obesity, insulin resistance, hypertension or atherosclerotic disease. Adiponectin may prevent the development of metabolic and atherosclerotic disorders. However, low adiponectin is currently considered a manifestation of insulin resistance and inflammation of fat tissue and a predictor of future cardiovascular events [19], [20]. A recent study showed that endoplasmic reticulum stress in the adipose tissue of obese mice down-regulated adiponectin production [21].

Adiponectin also plays an important role in myocardial remodeling [3], [15]. Animal studies have shown that adiponectin has a direct protective effect on myocardial hypertrophy [4], [22], [23]. Most clinical studies have also reported a negative relationship between adiponectin and LVMI [5]–[7], [24]. However, a positive relationship between the adiponectin level and LVMI has been reported in a few studies performed on patients who are receiving dialysis or have diabetes and who are at a higher risk of LVH than the general population. In the Jackson Heart Study, a positive relationship was also observed in subjects with hypertension and insulin resistance, which are well-known risk factors for LVH, whereas negative relationships were observed in the other subjects. Furthermore, the study was performed in an African American population, which has a higher prevalence of LVH relative to other ethnic groups [25]. Similarly, in our study, a positive relationship between the adiponectin level and LVMI was found in subjects at high risk of LVH, whereas no relationship or a negative relationship was found in subjects at low risk of LVH.

Our data demonstrate that there was a biphasic distribution of adiponectin level with increasing LVMI. A recent study in spontaneously hypertensive rats also showed a biphasic distribution of plasma adiponectin accompanying the progression of LVH [26]. The authors emphasized that adiponectin decreases with LVH progression and then increases after systolic dysfunction develops. However, in their data, LVM continuously increased as adiponectin level fluctuated biphasically. Their study was the first to propose a biphasic nature for the adiponectin level during the progression of LVH. To our knowledge, our work is the first epidemiological study reporting a similar trend.

The correlations between the adiponectin level and LVMI in the healthy subject group and the moderate to severe LVH group were subtle and insignificant, although a negative and a positive correlation, respectively, were expected. This may be related to the direct and indirect range restriction of LVMI, because the severity of LVH almost horizontally divided the range of LVMI [27]. Reducing the range of LVMI may affect the strength of the correlation between the adiponectin level and LVMI. The adiponectin level in the groups with no risk factor for LVH was lower than in the group with one risk factor, although we expected it to be higher. However, because the adiponectin level is influenced by age, gender, obesity and insulin resistance, and the association between the adiponectin level and LVMI is rather modest, the adiponectin levels could be different from the expected levels. In particular, unlike the healthy subject group, the group with no risk factor for LVH consisted of subjects younger than 50 years old, which most likely influenced the adiponectin levels.

### Signaling between the Myocardium and Adipose Tissue

Increased adiponectin has been frequently associated with both systolic and diastolic heart failure. The mechanism for this paradoxical increase in adiponectin in CHF is still unclear. The cardiac myocyte may be a source of adiponectin [22]. However, ectopic adiponectin secreted from the myocardium is unlikely to increase plasma adiponectin because myocardial adiponectin production is decreased in patients with CHF and in an animal model of LVH [28], [29].

Instead, increased adiponectin in CHF is thought to be a compensatory mechanism against left ventricular remodeling, which involves BNP [15]. Increased BNP in CHF enhances adiponectin production from adipose tissue [30]. There is a considerable overlap between LVH and diastolic heart failure with preserved systolic function, and BNP is a well-known marker of LVM, even in subjects without systolic dysfunction [31]. Subjects with hypertension, insulin resistance, obesity or high arterial stiffness are more likely to have elevated BNP than healthy subjects [32]–[34]. Therefore, as in patients with CHF, the underlying mechanism for the positive relationship between the adiponectin level and LVMI observed in subjects at high risk of LVH may be associated with increased BNP. However, because there are no available data for BNP levels, further studies are necessary to elucidate the involvement of BNP in the mechanism of the relationship between the adiponectin level and LVMI in subjects at high risk of LVH.

Currently, high adiponectin is thought to be a predictor of low risk for future cardiovascular events [19]. In contrast, in patients with CHF, high adiponectin is an indicator of higher cardiovascular mortality [13]. Our study showed that higher adiponectin could also be an indicator of more severe myocardial remodeling in subjects at high risk of LVH, even without overt CHF. The positive relationship between adiponectin and LVMI may also suggest a compensatory response of adiponectin to myocardial remodeling in subjects at high risk of LVH. Finally, regardless of the risk group of LVH, the adiponectin level explained less than 5% of the variability in LVMI. Currently, no data are available on the influence of high adiponectin on the survival of patients with LVH or with CHF. However, this weak relationship may explain why high adiponectin does not improve the survival of patients with CHF, despite all of the beneficial effects of adiponectin on the cardiovascular system [14].

### Study Limitations

The Yangpyeong Cardiovascular Cohort was located in Yangpyeong County, a rural area of the country with a rapidly developing economy. Because of its regional nature, the population was generally older and had a higher prevalence of insulin resistance and a higher proportion of females compared to the populations of previous studies. Therefore, our results may contain a referral bias. Our study is cross-sectional and thus does not contain outcome or survival data. A longitudinal study is necessary to determine whether adiponectin is a bystander or an active agent in myocardial remodeling. BNP levels were not investigated in our study. Because BNP has been suggested to be a regulatory signal between the myocardium and adipose tissue, a multiple regression analysis, including adiponectin and BNP, would be useful to reveal the influence of BNP on adiponectin production.

In conclusion, the relationship between adiponectin level and LVMI varies with the risk of LVH. In subjects at low risk of LVH, the adiponectin level negatively correlates with LVMI. In contrast, the adiponectin level is positively associated with LVMI in subjects at high risk of LVH. These two different relationships between adiponectin level and LVMI may indicate different mechanisms that influence adiponectin production by adipose tissue according to the risk of LVH. Further investigations are required to elucidate these mechanisms.

## References

[1] Wiecek A, Adamczak M, Chudek J (2007) Adiponectin–an adipokine with unique metabolic properties. Nephrol Dial Transplant 22: 981–988. 17234664 10.1093/ndt/gfl814

[2] Kadowaki T, Yamauchi T, Kubota N, Hara K, Ueki K, et al (2006) Adiponectin and adiponectin receptors in insulin resistance, diabetes, and the metabolic syndrome. J Clin Invest 116: 1784–1792. 16823476 10.1172/JCI29126 PMC1483172

[3] Park M, Sweeney G (2012) Direct effects of adipokines on the heart: focus on adiponectin. Heart Fail Rev. 10.1007/s10741-012-9337-8 22893246

[4] Shibata R, Ouchi N, Ito M, Kihara S, Shiojima I, et al (2004) Adiponectin-mediated modulation of hypertrophic signals in the heart. Nat Med 10: 1384–1389. 15558058 10.1038/nm1137 PMC2828675

[5] Paakko T, Ukkola O, Ikaheimo M, Kesaniemi YA (2010) Plasma adiponectin levels are associated with left ventricular hypertrophy in a random sample of middle-aged subjects. Ann Med 42: 131–137. 20166815 10.3109/07853890903449827

[6] Ebinc H, Ebinc FA, Ozkurt ZN, Dogru MT, Tulmac M, et al (2008) Impact of adiponectin on left ventricular mass index in non-complicated obese subjects. Endocr J 55: 523–528. 18469485 10.1507/endocrj.k07e-098

[7] Mitsuhashi H, Yatsuya H, Tamakoshi K, Matsushita K, Otsuka R, et al (2007) Adiponectin level and left ventricular hypertrophy in Japanese men. Hypertension 49: 1448–1454. 17420337 10.1161/HYPERTENSIONAHA.106.079509

[8] Hong SJ, Park CG, Seo HS, Oh DJ, Ro YM (2004) Associations among plasma adiponectin, hypertension, left ventricular diastolic function and left ventricular mass index. Blood Press 13: 236–242. 15581338 10.1080/08037050410021397

[9] Adamczak M, Blach A, Kolonko A, Szotowska M, Chudek J, et al (2011) Plasma adiponectin concentration and left ventricular hypertrophy in kidney transplant patients. Clin Transplant 25: 561–568. 20964715 10.1111/j.1399-0012.2010.01330.x

[10] Bidulescu A, Liu J, Musani SK, Fox ER, Samdarshi TE, et al (2011) Association of adiponectin with left ventricular mass in blacks: the Jackson Heart Study. Circ Heart Fail 4: 747–753. 21840935 10.1161/CIRCHEARTFAILURE.110.959742 PMC3218236

[11] Ayerden Ebinc F, Ebinc H, Derici U, Aral A, Aybay C, et al (2009) The relationship between adiponectin levels and proinflammatory cytokines and left ventricular mass in dialysis patients. J Nephrol 22: 216–223. 19384839

[12] Komaba H, Igaki N, Goto S, Yokota K, Takemoto T, et al (2007) Adiponectin is associated with brain natriuretic peptide and left ventricular hypertrophy in hemodialysis patients with type 2 diabetes mellitus. Nephron Clin Pract 107: c103–108. 17890872 10.1159/000108651

[13] Kistorp C, Faber J, Galatius S, Gustafsson F, Frystyk J, et al (2005) Plasma adiponectin, body mass index, and mortality in patients with chronic heart failure. Circulation 112: 1756–1762. 16157772 10.1161/CIRCULATIONAHA.104.530972

[14] Tamura T, Furukawa Y, Taniguchi R, Sato Y, Ono K, et al (2007) Serum adiponectin level as an independent predictor of mortality in patients with congestive heart failure. Circ J 71: 623–630. 17456982 10.1253/circj.71.623

[15] Shinmura K (2010) Is adiponectin a bystander or a mediator in heart failure? The tangled thread of a good-natured adipokine in aging and cardiovascular disease. Heart Fail Rev 15: 457–466. 20204504 10.1007/s10741-010-9159-5

[16] Yang YJ, Choi BY, Chun BY, Kweon SS, Lee YH, et al (2010) Dietary zinc intake is inversely related to subclinical atherosclerosis measured by carotid intima-media thickness. Br J Nutr 104: 1202–1211. 20487581 10.1017/S0007114510001893

[17] Weisell RC (2002) Body mass index as an indicator of obesity. Asia Pac J Clin Nutr 11 Suppl 8S681–684.

[18] Lang RM, Bierig M, Devereux RB, Flachskampf FA, Foster E, et al (2005) Recommendations for chamber quantification: a report from the American Society of Echocardiography's Guidelines and Standards Committee and the Chamber Quantification Writing Group, developed in conjunction with the European Association of Echocardiography, a branch of the European Society of Cardiology. J Am Soc Echocardiogr 18: 1440–1463. 16376782 10.1016/j.echo.2005.10.005

[19] Zhang H, Mo X, Hao Y, Huang J, Lu X, et al.. (2012) Adiponectin Levels and Risk of Coronary Heart Disease: A Meta-analysis of Prospective Studies. Am J Med Sci. 10.1097/MAJ.0b013e318262dbef 23123561

[20] Lu JY, Huang KC, Chang LC, Huang YS, Chi YC, et al (2008) Adiponectin: a biomarker of obesity-induced insulin resistance in adipose tissue and beyond. J Biomed Sci 15: 565–576. 18535923 10.1007/s11373-008-9261-z

[21] Mondal AK, Das SK, Varma V, Nolen GT, McGehee RE, et al (2012) Effect of endoplasmic reticulum stress on inflammation and adiponectin regulation in human adipocytes. Metab Syndr Relat Disord 10: 297–306. 22545589 10.1089/met.2012.0002 PMC3449395

[22] Amin RH, Mathews ST, Alli A, Leff T (2010) Endogenously produced adiponectin protects cardiomyocytes from hypertrophy by a PPARgamma-dependent autocrine mechanism. Am J Physiol Heart Circ Physiol 299: H690–698. 20622112 10.1152/ajpheart.01032.2009 PMC2944479

[23] Wang C, Li L, Zhang ZG, Fan D, Zhu Y, et al (2010) Globular adiponectin inhibits angiotensin II-induced nuclear factor kappaB activation through AMP-activated protein kinase in cardiac hypertrophy. J Cell Physiol 222: 149–155. 19780028 10.1002/jcp.21931

[24] Frankel DS, Vasan RS, D'Agostino RB Sr, Benjamin EJ, Levy D, et al (2009) Resistin, adiponectin, and risk of heart failure the Framingham offspring study. J Am Coll Cardiol 53: 754–762. 19245965 10.1016/j.jacc.2008.07.073 PMC2676793

[25] Lloyd-Jones D, Adams RJ, Brown TM, Carnethon M, Dai S, et al (2010) Heart disease and stroke statistics–2010 update: a report from the American Heart Association. Circulation 121: e46–e215. 20019324 10.1161/CIRCULATIONAHA.109.192667

[26] Zhou J, Fu M, Qian J, Jin X, Zhong C, et al (2012) Adiponectin through its biphasic serum level is a useful biomarker during transition from diastolic dysfunction to systolic dysfunction - an experimental study. Lipids Health Dis 11: 106. 22935137 10.1186/1476-511X-11-106 PMC3492043

[27] Thorndike RL (1947) Research problems and techniques. Washington, DC: U.S. Govt. print. off. viii, 163 pp.

[28] Liao Y, Xuan W, Zhao J, Bin J, Zhao H, et al (2010) Antihypertrophic effects of adiponectin on cardiomyocytes are associated with the inhibition of heparin-binding epidermal growth factor signaling. Biochem Biophys Res Commun 393: 519–525. 20152812 10.1016/j.bbrc.2010.02.039

[29] Skurk C, Wittchen F, Suckau L, Witt H, Noutsias M, et al (2008) Description of a local cardiac adiponectin system and its deregulation in dilated cardiomyopathy. Eur Heart J 29: 1168–1180. 18390538 10.1093/eurheartj/ehn136

[30] Tsukamoto O, Fujita M, Kato M, Yamazaki S, Asano Y, et al (2009) Natriuretic peptides enhance the production of adiponectin in human adipocytes and in patients with chronic heart failure. J Am Coll Cardiol 53: 2070–2077. 19477358 10.1016/j.jacc.2009.02.038

[31] Luchner A, Burnett JC Jr, Jougasaki M, Hense HW, Heid IM, et al (2000) Evaluation of brain natriuretic peptide as marker of left ventricular dysfunction and hypertrophy in the population. J Hypertens 18: 1121–1128. 10954005 10.1097/00004872-200018080-00018

[32] Sung SH, Wu TC, Huang CH, Lin SJ, Chen JW (2011) Prognostic impact of body mass index in patients undergoing coronary artery bypass surgery. Heart 97: 648–654. 21330312 10.1136/hrt.2010.211110

[33] Tekes S, Cikim AS (2007) The association of brain natriuretic peptide and insulin resistance in obesity-related hypertension. J Hum Hypertens 21: 546–550. 17392814 10.1038/sj.jhh.1002194

[34] Siervo M, Ruggiero D, Sorice R, Nutile T, Aversano M, et al (2010) Angiogenesis and biomarkers of cardiovascular risk in adults with metabolic syndrome. J Intern Med 268: 338–347. 20649935 10.1111/j.1365-2796.2010.02255.x

